# Digitale Technologien in der Pflege – Was können sie leisten?

**DOI:** 10.1007/s00103-024-03843-3

**Published:** 2024-02-07

**Authors:** Karin Wolf-Ostermann, Heinz Rothgang

**Affiliations:** 1 Institut für Public Health und Pflegeforschung, Universität Bremen, Grazer Str. 4, 28359 Bremen, Deutschland; 2 Leibniz-WissenschaftsCampus Digital Public Health, Bremen, Deutschland; 3 SOCIUM Forschungszentrum Ungleichheit und Sozialpolitik, Universität Bremen, Bremen, Deutschland

**Keywords:** Digitalisierung, Digitale Pflegetechnologien, Künstliche Intelligenz (KI), Pflege, Technik, Digitalization, Digital nursing technologies, Artificial intelligence (AI), Nursing, Technology

## Abstract

Digitale Pflegetechnologien gewinnen in der Langzeitpflege zunehmend an Bedeutung. Sie umfassen alle Technologien, die mittels Vernetzung und/oder Sensorik Prozesse und/oder Produkte verändern, und schließen künstliche Intelligenz, also Verfahren, Methoden und Algorithmen, um mittels Daten zu lernen und darauf aufbauend zielorientierte Handlungen zu ermöglichen, ein. Ihre Anwendung reicht von der Förderung professioneller Zusammenarbeit über Steuerung und Verwaltung, Wissenserwerb und -weitergabe, Interaktion und Beziehung bis zur körpernahen Pflege.

Digitale Pflegetechnologien haben das Potenzial, gleichzeitig die Qualität der Pflege zu erhöhen und die Arbeitsbedingungen in der Pflege zu verbessern. Allerdings stehen dem Hemmnisse auf verschiedenen Ebenen entgegen: Die Entwicklung dieser Technologien wird häufig von den technischen Möglichkeiten getrieben, sodass Produkte entstehen, die im Pflegealltag keinen konkreten Nutzen entfalten. Bei der Implementation wird nur die Bedienung geschult; es erfolgt aber keine Organisationsentwicklung zur systematischen Integration der Technologien in den Arbeitsalltag. Zudem fehlen hochwertige Evaluationen, die den tatsächlichen Nutzen im Arbeitsalltag abbilden, um so potenzielle Anwender:innen für die Technologie zu gewinnen. Schließlich ist die nachhaltige Finanzierung, insbesondere der Unterhaltung dieser Technologien, nicht gesichert.

Eine gelingende Digitalisierung in der Pflege setzt daher voraus, dass Technikentwickler:innen und -anwender:innen ebenso wie Politik und Wissenschaft gemeinsam diese Hemmnisse überwinden. Das impliziert, dass Pflegende von Anfang an in den Entwicklungsprozess einbezogen sind, aber auch dass Orte geschaffen werden, in denen die Wirkung digitaler Pflegetechnologien im tatsächlichen Versorgungsgeschehen evaluiert werden kann.

## Abstract (en)

Digital care technologies are becoming increasingly important in long-term care. They encompass all technologies that change processes and products by means of networking and/or sensor technology and include artificial intelligence, that is, processes, methods, and algorithms for learning by means of data and enabling meaningful decisions based on this. Their application ranges from the promotion of professional collaboration, control and management, knowledge acquisition and transfer, interaction and relationships to physical caregiving.

Digital care technologies have the potential to simultaneously increase the quality of care and improve working conditions in care. However, there are obstacles to this at various levels: The development of these technologies is often driven by technical possibilities, resulting in products that do not provide any concrete benefits in routine nursing care. During implementation, only the operator is trained; however, there is no organizational development for the systematic integration of these technologies into routine work. In addition, there is a lack of high-quality evaluations showing evidence of the actual benefits to routine work in order to attract potential users to these technologies. Finally, there is no sustainable financing, especially for the maintenance of these technologies.

Successful digitization in long-term care therefore requires that technology developers and users, as well as policymakers and scientists, jointly overcome these obstacles. This implies that caregivers are involved in the development process from the outset (co-creation) but also that spaces are created where the effect of digital care technologies can be evaluated in routine caregiving.

## Einleitung

Vor dem Hintergrund des demografischen Wandels, veränderter Familien- und Sorgestrukturen sowie eines damit verbundenen Personalmangels steht die Pflege in Deutschland vor großen Herausforderungen. Beispielhaft sei hier die Entwicklung in der Langzeitpflege genannt, wo sich die Zahl der Pflegebedürftigen von rund 5 Mio. im Dezember 2021 [1] auf rund 7,5–8 Mio. im Jahr 2050 erhöhen wird [2] – was die Notwendigkeit der Schaffung entsprechender Pflegekapazitäten impliziert. Bei Beibehaltung der derzeitigen Betreuungsrelationen resultiert hieraus allein in der Langzeitpflege ein Pflegepersonalmehrbedarf von rund 600.000 Personen im Zeitraum von 2020 bis 2050. Schon jetzt besteht aber ein Mangel an Pflegekräften, insbesondere an Pflegefachkräften: So konnten im Jahresdurchschnitt 2020/2021 über 17.000 Stellen für Fachkräfte in der Altenpflege und über 14.000 für Fachkräfte der Gesundheits- und Krankenpflege nicht besetzt werden [3]. Zudem müssten in der stationären Langzeitpflege mehr als 100.000 zusätzliche Stellen (in Vollzeit) geschaffen werden, damit eine Pflege nach dem aktuellem Pflegeverständnis möglich ist – so das Ergebnis der vom Bundesgesetzgeber beauftragten Studie zur Entwicklung und Erprobung eines Personalbemessungsverfahrens [4]. Auch in der Krankenhauspflege ist davon auszugehen, dass das neue Personalbemessungsverfahren, das derzeit entwickelt wird, höhere Stellenschlüssel generiert. Vor dieser sich weiter öffnenden Schere von Bedarfen und Angeboten wird der „Megatrend Digitalisierung“ auch in der Pflege vermehrt als Lösungsansatz diskutiert [5]. Dies zeigt sich in der Gesetzgebung ebenso wie in der Forschung.

So ist seit 2015 eine Reihe von Gesetzen zur Digitalisierung in Gesundheit und Pflege verabschiedet worden. Das am 29.12.2015 in Kraft getretene, auch als „E-Health-Gesetz“ bezeichnete „Gesetz für sichere digitale Kommunikation und Anwendungen im Gesundheitswesen“ (BGBl. I, 2015, S. 1408) vom 15.12.2015 hat die ersten Weichen für digitale Gesundheitsanwendungen gestellt. Es wurde gefolgt vom „Gesetz für eine bessere Versorgung durch Digitalisierung und Innovation (Digitale-Versorgung-Gesetz – DVG)“ vom 09.12.2019 (BGBl. I, 2019, S. 2562) zur Erweiterung der Telematik-Infrastruktur, dem „Gesetz zum Schutz elektronischer Patientendaten in der Telematikinfrastruktur (Patientendaten-Schutz-Gesetz – PDSG)“ vom 14.10.2020 (BGBl. I, 2020, S. 2115) mit Regelungen zur elektronischen Patientenakte (ePA) und zum E‑Rezept sowie dem „Krankenhauszukunftsgesetz“ (Gesetz für ein Zukunftsprogramm Krankenhäuser (Krankenhauszukunftsgesetz – KHZG)) vom 23.10.2020 (BGBl. I, 2020, S. 2208) zur Förderung und Evaluation der digitalen Infrastruktur in Krankenhäusern. Das „Gesetz zur digitalen Modernisierung von Versorgung und Pflege (Digitale-Versorgung-und-Pflege-Modernisierungs-Gesetz – DVPMG)“ vom 03.06.2021 (BGBl. I, 2021, S. 1309) befasst sich mit neuen digitalen Anwendungen insbesondere auch in der Pflege (z. B. digitale Pflegeanwendungen (DiPA) und digitale Inhalte der Pflegeberatung). Der vorläufige Abschluss ist das „Gesetz zur Unterstützung und Entlastung in der Pflege (Pflegeunterstützungs- und -entlastungsgesetz – PUEG)“ vom 23.06.2023 (BGBl. I, 2023, Nr. 155), das neben der stärkeren Fokussierung auf digitale Anwendungen und Vernetzungen in der Pflege insbesondere auch die Etablierung eines Kompetenzzentrums Digitalisierung und Pflege thematisiert.

Auch in der akademischen Pflege und der Pflegeforschung sind die Themen von Technik und Digitalisierung in der Pflege zunehmend sichtbar. Zum einen werden in Studiengängen entsprechende Module integriert bzw. entsprechende Studiengänge (z. B. „Pflegeinformatik“) entwickelt und entsprechend spezifisch zugeschnittene Professuren eingerichtet. Zum anderen wurden in den vergangenen Jahren pflegespezifische Forschungscluster gefördert: Den Anfang machten im Rahmen der Förderlinie „Zukunft der Pflege“ des Bundesministeriums für Bildung und Forschung (BMBF) ab dem Jahr 2017 das Pflegeinnovationszentrum (PIZ; [6]) sowie die Pflegepraxiszentren (PPZ), in denen soziale und technische Innovationen in der Pflege unter realistischen Bedingungen erforscht und weiterentwickelt werden.^1^ Ziele sind hierbei insbesondere, formell und informell Pflegende zu entlasten, die Pflegequalität zu steigern und die Lebensqualität von Menschen mit Pflegebedarf zu verbessern. Im Rahmen der Förderlinie „Pflege und Robotik“ hat das Bundesministerium für Bildung und Forschung (BMBF) ab 2020 dann 10 interdisziplinäre Projektverbünde zur Entwicklung, Erprobung und Evaluation von Robotik für die Pflege gefördert.^2^ Auch hier standen die Ziele einer Entlastung von formell und informell Pflegenden, die Steigerung der Pflegequalität sowie die Förderung der Selbstständigkeit und des Wohlbefindens von Menschen mit Pflegebedarf im Vordergrund. Die Förderlinie „Technologiegestützte Innovationen für Sorgegemeinschaften zur Verbesserung von Lebensqualität und Gesundheit informell Pflegender (PAZ)“ des BMBF fokussiert die Erforschung und Entwicklung von interaktiven Technologien für informell Pflegende.^3^ Weitere Förderlinien sind „Repositorien und KI-Systeme im Pflegealltag nutzbar machen“^4^ sowie – diesmal vom Bundesministerium für Gesundheit (BMG) gefördert – „Künstliche Intelligenz in der professionellen Langzeitpflege“^5^ mit dem Ziel der Entlastung des pflegerischen Personals bei der Versorgungsplanung und der Pflegedokumentation. Daneben wird die Einführung digitaler Pflegetechnologien in einer Vielzahl weiterer Einzelförderungen in zum Teil durchaus beachtlichem Umfang gefördert – hierzu zählen beispielsweise auch Kompetenzzentren zum Thema „Pflege und Digitalisierung“ in den einzelnen Bundesländern.

Schließlich hat der Gesetzgeber diverse Fördertöpfe (auf Bundes- und Landesebene) eingerichtet, um die Einführung digitaler Pflegetechnologien zu finanzieren, insbesondere den § 8 Abs. 8 SGB XI, der allen ambulanten und stationären Pflegeeinrichtungen die Finanzierung von Anschaffungen digitaler oder technischer Ausrüstung sowie der damit verbundenen Schulungen in Umfang von bis zu 12.000 € pro Einrichtungen ermöglicht.

Dass das Thema „Digitalisierung“ auch in der Pflege angekommen ist, steht damit außer Frage. Aber trägt Digitalisierung tatsächlich dazu bei, die Herausforderungen, denen sich die Pflege stellen muss, besser zu meistern? Zur Beantwortung dieser Frage folgt der vorliegende Artikel einem Dreischritt: Nach einer Übersicht über die Anwendungsspektren wird die Evidenz zum Nutzen digitaler Technologien in der Pflege resümiert. Anschließend werden die Chancen, Risiken und Hemmnisse bei der Digitalisierung diskutiert. Abschließend werden die Weichenstellungen benannt, die notwendig sind, damit Digitalisierung in der Pflege die Versorgungssituation der zu Pflegenden und die Arbeitssituation der Pflegepersonen verbessern kann.

## Zum Stand der digitalen Technik in der Pflege

„Digitalisierung bedeutet die Verwendung von Daten und algorithmischen Systemen für neue oder verbesserte Prozesse, Produkte und Geschäftsmodelle“ [7]. Krick [8, S. 4 f.] definiert „digitale Pflegetechnologien“ als „Hardware und/oder Software(‑Erzeugnisse), die anhand von bestimmten Regeln Entscheidungen treffen. Dies beinhaltet insbesondere informationstechnisch vernetzte und/oder mit Sensorik ausgestattete (digitale) Entwicklungen, die

- pflegende Personen oder die Organisation in ihrem pflegerischen Handeln unterstützen,
- dazu beitragen, dass Personen so in ihrer sozialen, physischen und/oder psychischen Eigenständigkeit unterstützt werden, dass auf direkte pflegerische Unterstützung vor Ort verzichtet werden kann,
- die Ausbildung, den Wissenstransfer und die Kompetenzentwicklung von Personen unterstützen.“

Digitale Pflegetechnologien können somit auch pflegerische Unterstützung substituieren oder die Aus‑, Fort- und Weiterbildung von Pflege(fach)personen unterstützen [9]. Ausschließlich mechanische Hilfsmittel, Technologien der medizinischen Diagnostik und invasive Technologien sowie ausschließlich auf Spaß abzielende Spiele sind damit nicht erfasst. Tab. 1 gibt einen Überblick über eine mögliche Kategorisierung von Anwendungsfeldern digitaler Technologien. Die Beispiele und deren Zuordnung zu den Anwendungsbereichen sind den Ergebnissen des Gutachtens „Digitale Technologien für die Pflege (GuDiT)“ [10] entnommen.

**Table 1:** 

*Professionelle Zusammenarbeit*	Patient:innenportale und Pflegeportale
	Elektronische Partient:innenakten
	Televisite
*Steuerung und Verwaltung*	Digitales Patient:innenmanagement
	Hausnotrufsysteme
	Dokumentation mit Spracheingabe
	Asset Tracking, RFID (Radio-Frequency Identification) und IoT (Internet of Things)
	Digitale Teammeetings
	Digital Companion
	Digitale Dienst- und Tourenplanung
	Intelligente Software für Tourenplanung
	Digitale Pflegedokumentation
*Wissenserwerb und -weitergabe*	Simulationsbasiertes Lernen: Skills Lab
	Digitale Teammeetings
	E‑Learning-Software
	Telepräsenzsysteme
*Interaktion und Beziehung*	Soziale Roboter
	Digitale Aktivitätsspiele
	Sensorisch stimulierende Assistenzsysteme
	Kommunikations-Apps
*Körpernahe Pflege*	Aktives Exoskelett
	Intelligentes Besteck
	Digitale Sturzprophylaxe und -erkennung
	Digitale Personenortungs- und Lokalisierungssysteme
	Intelligente Inkontinenzprodukte
	Intelligente Matratzen
	Intelligente Pflaster
	Serviceroboter

Technik und Pflege gehören ebenso wie Digitalisierung und Pflege im Versorgungsalltag bereits vielfach untrennbar zusammen. Eine 2020 vom IGES-Institut veröffentlichte Umfrage zum Technikeinsatz und zum aktuellen Technisierungsgrad von Pflegeeinrichtungen der Langzeitpflege zeigt beispielsweise eine stärkere Nutzung in stationären Pflegeeinrichtungen im Vergleich zu ambulanten Diensten [11]. Genutzt werden vor allem Technologien zur betrieblichen Organisation und Verwaltung (z. B. Rechnungswesen) vor Technologien zur direkten pflegerischen Versorgung (z. B. Lifter, Sturzerfassung, Notrufe). Assistenz- und Serviceroboter sind nur punktuell im Einsatz und auch ein digitaler Datenaustausch mit anderen Leistungserbringern findet nur selten statt.

Das Pflegeinnovationszentrum (PIZ) zeigte ebenfalls 2020 im Rahmen einer Querschnittstudie zu Bedarfen und Erfahrungen mit neuen Technologien im pflegerischen Arbeitsalltag auf, dass rund 95 % (*n* = 1018) der befragten in der Pflege tätigen Personen über praktische Erfahrungen mit neuen Technologien verfügen [12]. Hierzu gehören in erster Linie „geläufige/tradierte“ Technologien (elektronische Pflegedokumentation, Dienst- und Tourenplanung, Aufstehhilfen). Eher selten werden Erfahrungen mit Sensorik (19 %), Interaktions- und Simulationstechnologien (9 %) oder Robotik (7 %) benannt. Settingspezifische Trends waren hierbei nicht erkennbar. Sowohl Pflegefachpersonen als auch Führungskräfte zeigten eine grundsätzlich positive Einstellung gegenüber neuen Technologien und sahen in dem Einsatz deutliche Arbeitserleichterungen, Zeit- und Effizienzgewinne sowie das Potenzial einer verbesserten pflegerischen Versorgung. Die Sorge, durch neue Technologien einer stärkeren Kontrolle ausgesetzt zu sein oder die eigene Arbeit damit „überflüssig“ zu machen, wurde hingegen deutlich verneint [12].

**Figure 1:**
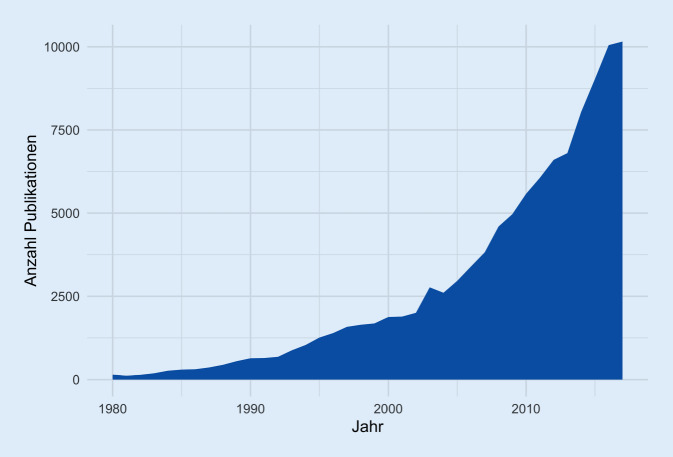

Die steigende Bedeutung von Technik in der Pflege zeigt sich noch deutlicher bei der Analyse der einschlägigen Publikationen. Eine 2018 durchgeführte systematische Literaturrecherche verdeutlicht den exponentiellen Anstieg der Zahl der entsprechenden Publikationen im Zeitverlauf.^6^ 2017 lag der jährliche internationale Publikationsoutput bereits bei mehr als 10.000 (Abb. 1).

**Figure 2:**
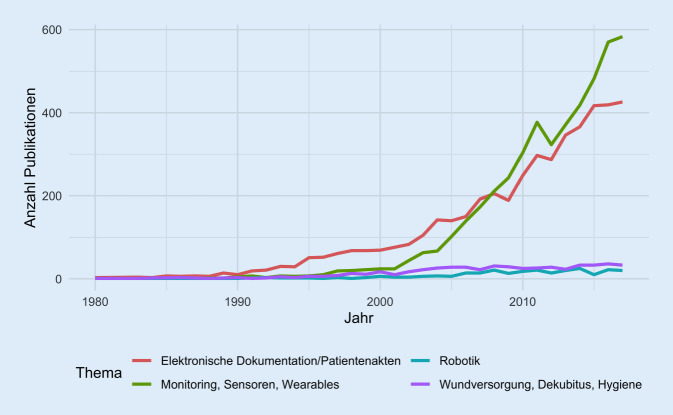

Thematisch werden dabei vor allem elektronische Dokumentationen/Patientenakten sowie Monitoring, Sensoren und Wearables beforscht (Abb. 2).

## Akzeptanz, Wirksamkeit und Effizienz digitaler Pflegetechnologien

Zur Akzeptanz, Wirksamkeit und Effizienz digitaler Pflegetechnologien liegt ein umfassender Review aus dem Jahr 2019 vor. Diese Arbeit von Krick et al. [9] zeigt, dass Studien zu Akzeptanz und Wirksamkeit dabei deutlich häufiger vertreten sind als Studien zur Effizienz von Technologien, die selten zu finden sind. Akzeptanz und Wirksamkeit wurden am häufigsten für Informations- und Kommunikationstechnologien (IKT), Roboter und Sensoren bzw. elektronische Dokumentationssysteme (EHR/EMR und HIS) untersucht. Bezüglich der Anwendungssettings sind die meisten der einbezogenen Technologien für die Krankenhauspflege, die informelle Pflege zu Hause und die stationäre Langzeitpflege bestimmt. Nur wenige Studien befassen sich mit der formellen Pflege zu Hause und kaum eine mit sektorübergreifender Pflege. Auch bezüglich der Zielgruppen zeigen sich Unterschiede: Die häufigste Zielgruppe sind pflegebedürftige Menschen, gefolgt von formell Pflegenden. Technologien für informelle Pflegekräfte und Kinder werden relativ selten erforscht. Die berichteten Trends unterscheiden sich für einige der Technologiekategorien.

Die meisten Studien weisen ein niedriges Evidenzniveau auf. So werden zur Bewertung von Akzeptanz und Wirksamkeit am häufigsten experimentelle Designs mit kleinen Fallzahlen und ohne Kontrollgruppen verwendet. Studiendesigns mit hohem Evidenzgrad finden sich in der Literatur am ehesten für IKT, Roboter und E‑Learning. Insgesamt fehlen hochwertige Studien allerdings weitgehend, sodass hier ein dringender Forschungsbedarf zu konstatieren ist. Dies gilt umso mehr, als ein Zusammenhang zwischen Evidenzniveau und der konstatierten Wirksamkeit festzustellen ist und das Ausmaß der erkannten Wirksamkeit mit dem Evidenzniveau der Studien sinkt [14].

Explizite Studien zu Effizienz sind sehr selten. Sie umfassen Pflege- und Gesundheitsinformationstechnologien wie Hilfsmittel, IKT, Sensoren und Robotik. Auch hier ist der Grad der Evidenz meist gering und die Studiengrößen sind oft klein. Kaum eine Technologie ist intensiv genug erforscht worden, um schlüssige Ergebnisse zu liefern. Für die meisten Technologiebereiche gibt es keine Studien auf hohem Evidenzniveau. Heterogene Ergebnisse in einigen Bereichen deuten darauf hin, dass die Auswirkungen stark von der Art und Weise und dem spezifischen Kontext, in dem die Technologien eingeführt werden, abhängen können [14].

Forschung zu digitalen Technologien für die Pflege wird weltweit betrieben, jedoch zeigen sich länderspezifische Besonderheiten in den Veröffentlichungen. Im Fokus der Forschung aus den USA und China finden sich vorrangig Publikationen zu IKT (speziell EHR/EMR und HIS), wohingegen Publikationen aus Australien und Neuseeland oft robotische Technologien zum Thema haben. Analysierte Settings sind überwiegend Krankenhäuser und die stationäre Langzeitpflege. Zielgruppen sind primär Personen mit Pflege- und Unterstützungsbedarf. Hier richtet sich ein großer Teil explizit an Menschen mit Demenz – sowie professionell Pflegende [14].

## Künstliche Intelligenz (KI) in der Pflege

Der Einsatz von „künstlicher Intelligenz“ (KI) als neuester Zweig digitaler Pflegetechnologien gewinnt zunehmend an Aufmerksamkeit. KI wird dabei als Sammelbegriff für alle Klassen von Verfahren, Methoden und Algorithmen verwendet, die mittels Daten lernen und darauf aufbauend intelligente, zielorientierte Handlungen ermöglichen. Häufig verwendete KI-Systeme sind maschinelles Lernen, aber auch Expertensysteme und hybride Systeme, die verschiedene KI-Fähigkeiten durch die Integration von maschinellem Lernen und Expertensystemen kombinieren (vgl. auch [15]). Derzeit existiert jedoch keine generelle, allgemein anerkannte Klassifikation von gesundheits- oder pflegespezifischen KI-Teilgebieten [16].

Die verfügbare Literatur zeigt, dass KI in der Pflege ein stark wachsendes Thema ist, das zunehmend Aufmerksamkeit findet. Ein Review aus dem Jahr 2021 zeigt jedoch, dass die Publikationen fast ausschließlich der Grundlagenforschung zuzurechnen sind und weniger als 10 % tatsächlich Anwendungen in der Praxis beschreiben oder zumindest Daten aus dem praktischen Versorgungsalltag einsetzen [17]. Zieldimensionen bzw. Zweck des KI-Einsatzes sind beispielsweise das Erkennen/Monitoring von Aktivitäten bzw. des Gesundheitsstatus, ein Alarmmanagement (z. B. Sturzerkennung), die Vorhersage von Dekubitusrisiken, Schmerzassessment oder planerische Aufgaben (z. B. Touren- oder Dienstplanung). Zielgruppen sind zu etwa gleichen Teilen professionell Pflegende und Pflegebedürftige, informell Pflegende finden sich deutlich seltener – oftmals wird in der Literatur jedoch auch keine konkrete Zielgruppe ausgewiesen. Einsatzgebiete sind am häufigsten das Krankenhaus, darauf folgen alleinlebende Personen (in der eigenen Häuslichkeit). Ambulant pflegerische oder stationäre Settings werden eher selten benannt.

Im Rahmen einer vom BMBF geförderten Studie zur Analyse von Bedürfnissen, Anwendungsszenarien, Anforderungen, Erleichterungen und Barrieren für Forschungs- und Entwicklungsprojekte im Kontext von KI in der Pflege zeigte sich, dass aus Sicht von Stakeholdern aus Pflegepraxis, Pflegemanagement, Pflegewissenschaft, Pflegebildung, Informatik sowie Angehörigenvertreter:innen vor allem Bedarfs- und Anwendungsszenarien der Mikro- und Mesoebene der Pflege benannt wurden. Die diskutierten Bedarfe und Anwendungsschwerpunkte bezogen sich auf der Mikroebene pflegerischen Handelns auf eine Unterstützung durch KI in individuellen Handlungs- und Entscheidungssituationen (z. B. direkte, individuelle Pflegesituation mit Beteiligung einer pflegebedürftigen und einer pflegenden Person), auch auf die evidenzbasierte Auswahl von geeigneten Pflegemaßnahmen oder die Einbindung weiterer Berufsgruppen in den Versorgungsprozess. Auf der Mesoebene standen pflegerisches Handeln und Planen in Pflegeeinrichtungen im Vordergrund [18].

Ein bereits 2021 veröffentlichtes Anwendungsszenario der „Plattform Lernende Systeme“ (PLS)^7^ zeigt ebenfalls Möglichkeiten auf, wie KI-gestützte Assistenzsysteme pflegebedürftige Menschen sowie ihre formell und informell Pflegenden in der nahen Zukunft ggf. unterstützen können. Eine qualitative Studie der Plattform Lernende Systeme mit Gesundheitsfachkräften aus den Bereichen Pflege und Medizin aus dem Jahr 2023 stellte fest, dass Beschäftigte im Gesundheitswesen grundsätzlich aufgeschlossen gegenüber dem Einsatz von KI sind und dieser auch als entlastend angesehen wird [31]. Damit der Einsatz von KI den Versorgungsalltag verbessern kann, werden jedoch technische und organisatorische Veränderungen im stationären und ambulanten Arbeitsalltag als notwendig angesehen. Wichtigste Ziele der Anwendung werden in einer verbesserten Versorgungsqualität für Patientinnen und Patienten gesehen sowie in einer Entlastung von Gesundheitsfachkräften.

## Chancen, Risiken und Herausforderungen

Um den bereits in der Einleitung konstatierten Herausforderungen zu begegnen, bietet eine vermehrte Einbindung digitaler Lösungen in die pflegerische Versorgung erhebliche Potenziale [19, 20]. Diese Überzeugung zeigt sich auch in Befragungen von Pflege(fach)personen, die regelmäßig Ideen für technische Unterstützungen in den Problemfeldern des Arbeitsalltags benennen [12, 15]. Allerdings fehlt es derzeit an ausreichendem Wissen über den tatsächlich realisierbaren Nutzen von technischen/digitalen Lösungen im Versorgungsalltag [14, 17, 21, 22]. An dieser Stelle ist erheblicher Forschungsbedarf zu konstatieren.

Gleichzeitig sind aber auch Risiken digitaler Pflegetechnologien zu benennen. Zentral ist hier die Frage, ob vorhandene Ungleichheiten durch unterschiedlichen Zugang zu digitalen Pflegetechnologien noch verstärkt werden (Digital Divide) und ob z. B. digitale Kommunikation letztlich sogar zu einer Vereinsamung führen kann [23]. Bei der Einführung neuer Technologien muss deshalb immer sichergestellt sein, dass die menschliche Komponente einbezogen bleibt und ethische und soziale Fragestellungen ausreichend diskutiert werden [24]. Technische und digitale Anwendungen sind als ein Hilfsmittel unter anderen zu verstehen, sie können und sollen persönliche Zuwendung nicht ersetzen.

Zentrale Herausforderung bei der Realisierung der positiven Potenziale digitaler Pflegetechnologien ist deren Implementation in den Arbeitsalltag. Pflege ist immer durch die Interaktion von Pflegenden, zu Pflegenden und technologischen Assistenzen innerhalb von organisatorischen Kontexten geprägt. Die Implementation digitaler Pflegetechnologien in ein solches soziotechnisches System ist daher ein komplexer Prozess, der eine Vielzahl von Aspekten zu berücksichtigen hat und nur interdisziplinär im Zusammenspiel von Technikentwickler:innen und (formell oder informell) Pflegenden sowie ggf. auch Menschen mit Pflegebedarf zu lösen ist [25]. Hierbei darf es nicht darum gehen, dass technologische Entwicklungen versuchen, andere Beteiligte „abzuholen“, vielmehr müssen gemeinsam sinnvolle Lösungen für konsentierte Probleme erarbeitet werden.

Grundsätzlich lassen sich die Voraussetzungen für eine gelingende Einbindung technischer/digitaler Lösungen an 4 Bereichen festmachen:

- *Technische Voraussetzungen:* Hierzu gehören beispielsweise das Vorhandensein einer ausreichenden digitalen Infrastruktur (z. B. ein leistungsstarkes WLAN), aber auch die Operationalisierung pflegerischen Handels und das Explizieren von bisher impliziten Entscheidungen in der Pflege, der Aufbau von Datenbanken bzw. eine Übersicht zu Datenquellen und öffentlich zugänglichen Datensammlungen. Gerade im Bereich von KI-Anwendungen ist die Verfügbarkeit von ausreichenden und qualitativ hochwertigen Daten ein wesentlicher gestaltender (oder limitierender) Faktor. Anforderungen an die Nutzbarmachung von Daten in der Pflege für die Entwicklung von KI-Systemen fehlen derzeit noch.
- *Funktionale Voraussetzungen:* Hierunter ist zu verstehen, dass für die jeweilige technische Lösung ein klarer pflegerischer oder auch individueller Nutzen nachweisbar sein muss bzw. bereits nachgewiesen wurde – inklusive der Abwägung ethischer und sozialer Aspekte. Insbesondere für KI-basierte Lösungen sollte das Prinzip „human in the loop/middle“^8^ im Vordergrund stehen, sodass in der Zusammenarbeit von Mensch und Maschine bestmögliche Ergebnisse erzielt werden und diese auch nachvollziehbar bleiben.
- *Organisationsbezogene Voraussetzungen:* Eine erfolgreiche Entwicklung und Implementierung (digitaler) Technologien erfordern nicht nur passgenaue rechtliche Rahmenbedingungen und eine ausreichende Finanzierung, sondern auch den Einbezug in bestehende Arbeitsabläufe und eine entsprechende Organisations(weiter)entwicklung, ggf. die Schaffung eigener Koordinationsstellen und die Qualifikation aller Beteiligten (z. B. durch Einbezug in Ausbildung und/oder Studium).
- *Professionsbezogene Voraussetzungen:* Nicht zuletzt ist es erforderlich, dass in der Pflege eine Erweiterung des professionellen Selbstverständnisses erfolgt und ein aktiver Kompetenzerwerb in Bezug auf technische/digitale Anwendungen selbstverständlich wird, sodass darauf aufbauend eine partizipative Entwicklung und Anwendung neuer (digitaler) Technologien erfolgen können. Pflegefachpersonen müssen maßgeblich an der Entwicklung von digitalen Technologien beteiligt werden und in der Lage sein, die Bedarfe ihres Berufsfeldes für Technikentwicklung zu benennen.

Erste – auch praxisbezogene – Ansätze für die Implementierung von Technologien liegen bereits vor, so etwa der „Implementierungsleitfaden aus und für die Praxis“, der konkrete Erfahrungen und anwendungsbezogenes Know-how umfasst und als Unterstützungsangebot für alle Stakeholder wesentliche Bedingungen, Gelingensfaktoren und Fragen einer Implementierung aus Sicht der implementierenden Organisation beschreibt (siehe auch [26]). Umfassender stellen die NASSS-CAT-Tools (Complexity Assessment Tools; [27]) und deren deutschsprachige Übersetzung [28] sowie die Adaption NASS-CAT‑D [29] verschiedene Instrumente zur Analyse der Komplexität und zur Planung von Maßnehmen zur Komplexitätsreduktion zur Verfügung und erleichtern das Management von Projekten zur Implementierung von Gesundheitstechnologien sowie zur vertiefenden Analyse von Veränderungsprozessen.

## Entfaltung der Potenziale von Pflegetechnologien

Es lässt sich schon jetzt konstatieren, dass Technik und neue Technologien kaum mehr aus der Pflege wegzudenken sind. Digitale Pflegetechnologien haben ein großes Potenzial, die Pflegequalität zu erhöhen, die Arbeitsbedingungen der Pflegekräfte zu verbessern und Arbeitszeit zu gewinnen [8]. Auch die Aufgeschlossenheit Pflegender gegenüber neuen, digitalen Technologien ist höher als vielfach gedacht – jedoch nur, wenn diese Technologien partizipativ entwickelt und eingeführt werden und wenn sie aus Sicht der Pflegenden reale Probleme adressieren [12]. Allerdings gibt es derzeit wenig gute Evaluationen, die den Nutzen digitaler Pflegetechnologien tatsächlich nachweisen [9, 14].

Was muss aber geschehen, damit digitale Pflegetechnologien ihr Potenzial entfalten und zu „guter Pflege“ beitragen können?

Die Einführung von Gesundheitstechnologien scheitert oft an einer unzureichenden Berücksichtigung der Komplexität der Einführungsprozesse. Neue Technologien werden zudem häufig aus Perspektive der Technik entwickelt und gehen am Bedarf der Nutzer:innen vorbei [5]. Weiterhin fehlt eine systematische Finanzierung für Pflegeinnovationen [5]. Zwar werden Projekte zur Einführung und Erprobung von Technologien gefördert, aber diese Förderung bezieht sich in der Regel nur auf die Anschaffung, nicht aber auf den Unterhalt der Technologien. Fehlende Finanzierungsmöglichkeiten wurden in der bereits genannten Umfrage von IGES als größtes Hemmnis für eine (stärkere) Nutzung benannt – auch wenn die Notwendigkeit eines größeren Techniknutzens in der Zukunft gesehen wird [11]. Zudem fehlt es an einer ausreichenden (Weiter‑)Bildung zum Einbezug der neuen Technologien in den Pflegealltag, da sich Schulungen häufig nur auf die Benutzung der Technik, aber nicht auf deren routinemäßige Integration in den Arbeitsalltag beziehen [5]. Schließlich gibt es derzeit keine regelhaften Orte, an denen Technologien im Alltag erprobt und evaluiert werden (können).

Vor diesem Hintergrund erfordert eine gelingende Digitalisierung in der Pflege Anstrengungen aller Beteiligten:

Technikentwickler müssen aufhören, aus ihrer eigenen Logik „Lösungen“ zu konstruieren, für die sie anschließend ein „Problem“ suchen. Dies impliziert, dass die Entwicklung digitaler Technik partizipativ erfolgen muss, damit nicht nur auf die Benutzerfreundlichkeit (Usability) geschaut wird, sondern auch darauf, ob für die Anwender:innen ein erkennbarer praktischer Nutzen entsteht.

Politik muss es Pflegeeinrichtungen ermöglichen, in digitale Technologie zu investieren. Diese Investitionen können aus Gewinnen finanziert werden, wenn ein – begrenzter – Gewinn regelmäßig in Vergütungsverhandlungen als Kostenposten anerkannt wird. Alternativ könnte ein Digitalisierungsbudget vorgesehen werden, über das Einrichtungen verfügen. Wichtig ist dabei, dass daraus nicht nur die Anschaffung, sondern auch der Unterhalt der Technologien – einschließlich der Finanzierung von Personen, die für die Aufrechterhaltung des Regelbetriebs zuständig sind – finanziert werden können.

Einrichtungen benötigen eine systematische Digitalisierungsstrategie, die über einen längeren Zeitraum eine sukzessive Digitalisierung – ausgehend von basalen Voraussetzungen wie einem leistungsfähigen WLAN – zu zunehmend komplexeren Anwendungen führt. Die isolierte Anschaffung einzelner digitaler Hilfsmittel ohne Gesamtstrategie ist dagegen nicht zielführend. Sinnvoll wäre in diesem Zusammenhang auch die Schaffung verstetigter Beratungszentren, die übergreifend eine Beratung sowohl für Technikentwickler:innen als auch für potenzielle Techniknutzer:innen und Risikokapitalgeber:innen leisten.

Die Wissenschaft schließlich muss hochwertige Evaluationen digitaler Pflegetechnologien – einschließlich ökonomischer Evaluationen, für die es entsprechende Frameworks gibt [30] – im Alltagsbetrieb zur Verfügung stellen. Notwendig hierfür sind Orte, an denen systematisch die Erbringung von Versorgungsleistungen mit Forschung und Lehre verknüpft wird – und nicht lediglich Reallabore und Showrooms, die die Tücken des Alltags systematisch unberücksichtigt lassen.

## Footnotes

1 https://www.cluster-zukunft-der-pflege.de/; letzter Zugriff: 27.08.2023.

2 https://www.pflege-und-robotik.de/; letzter Zugriff: 27.08.2023.

3 https://www.interaktive-technologien.de/foerderung/bekanntmachungen/paz; letzter Zugriff: 19.10.2023.

4 https://www.interaktive-technologien.de/foerderung/bekanntmachungen/kip/; letzter Zugriff 27.08.2023.

5 https://www.bundesgesundheitsministerium.de/ministerium/ressortforschung/handlungsfelder/forschungsschwerpunkte/kuenstliche-intelligenz/etap.html/; letzter Zugriff am 27.08.2023.

6 Die Recherche wurde in den Datenbanken Medline, Scopus, CINAHL, Cochrane Library, ACM Digital Library, IEEE Xplore und The Collection of Computer Science Bibliographies durchgeführt.

7 https://www.plattform-lernende-systeme.de/ki-zur-unterstuetzung-in-der-pflege.html; letzter Zugriff am 22.10.2022.

8 „Human in the loop/middle“: Interaktion zwischen Mensch und künstlicher Intelligenz (KI) zwecks Training und Optimierung eines KI-Systems sowie Ausrichtung des Einsatzes von KI auf den Menschen („menschzentrierte KI“).
